# A Multimodal Protein Representation Framework for Quantifying Transferability Across Biochemical Downstream Tasks

**DOI:** 10.1002/advs.202301223

**Published:** 2023-05-30

**Authors:** Fan Hu, Yishen Hu, Weihong Zhang, Huazhen Huang, Yi Pan, Peng Yin

**Affiliations:** ^1^ Guangdong‐Hong Kong‐Macao Joint Laboratory of Human‐Machine Intelligence‐Synergy Systems Shenzhen Institute of Advanced Technology Chinese Academy of Sciences Shenzhen 518055 China

**Keywords:** protein representation, multi‐modal, transferability, downstream tasks

## Abstract

Proteins are the building blocks of life, carrying out fundamental functions in biology. In computational biology, an effective protein representation facilitates many important biological quantifications. Most existing protein representation methods are derived from self‐supervised language models designed for text analysis. Proteins, however, are more than linear sequences of amino acids. Here, a multimodal deep learning framework for incorporating ≈1 million protein sequence, structure, and functional annotation (MASSA) is proposed. A multitask learning process with five specific pretraining objectives is presented to extract a fine‐grained protein‐domain feature. Through pretraining, multimodal protein representation achieves state‐of‐the‐art performance in specific downstream tasks such as protein properties (stability and fluorescence), protein‒protein interactions (shs27k/shs148k/string/skempi), and protein‒ligand interactions (kinase, DUD‐E), while achieving competitive results in secondary structure and remote homology tasks. Moreover, a novel optimal‐transport‐based metric with rich geometry awareness is introduced to quantify the dynamic transferability from the pretrained representation to the related downstream tasks, which provides a panoramic view of the step‐by‐step learning process. The pairwise distances between these downstream tasks are also calculated, and a strong correlation between the inter‐task feature space distributions and adaptability is observed.

## Graphical abstract

In this work, a comprehensive protein representation framework integrating sequence, structure, and function is introduced. This model excels in diverse applications. A novel spatial metric is also presented, assessing the dynamic transferability of pretrained protein representations to downstream tasks, offering insights into the step‐by‐step learning process.

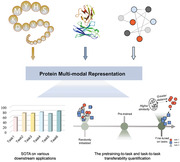

## 1 Introduction

Proteins are the building blocks of life, carrying out fundamental functions in biology. A natural protein, which is made up of a linear sequence of amino acids linked by peptide bonds, folds into its 3D or tertiary structure to perform its biological function. Understanding a protein from its sequence, structure, and function is one of the greatest scientific challenges of the 21st century, as it is essential for elucidating the mechanisms of disease and therapeutic development. In recent years, the exponential growth in available protein data (e.g., sequences, structures, and functional annotations) has provided fertile resources for studying proteins using computational methods, particularly artificial intelligence.^[^ ^1^ ^]^

Artificial intelligence techniques have become mainstream in this area, and they usually start with representation learning.^[^ ^2^ ^]^ A representation is, at its core, a distillation of raw data into a high‐level, typically low‐dimensional space that captures the underlying features of the complex raw data. This representation, usually a numerical vector or array, can then be utilized for a variety of purposes, including task‐specific prediction (e.g., binding prediction) and interpretable exploration. A good representation can be greatly helpful for improving the performance of a model.^[^ ^3^, ^4^, ^5^, ^6^, ^7^ ^]^ Recently, self‐supervised pretrained language models have emerged as a powerful paradigm for learning efficient representations from raw data in various fields, such as natural language processing (NLP) and computer vision (CV).^[^ ^4^, ^8^ ^]^ The goal of a language model is to learn underlying semantics from large text corpora based on the distributional hypothesis: words that appear in similar contexts may have similar meanings.

Proteins can be regarded as the natural language of biology, written with multiple amino acid words,^[^ ^1^ ^]^ and thus, NLP language models can be well applied. The corresponding protein representations have shown excellent performance in a variety of protein‐related downstream applications, such as predictions of protein stability and mutational effects.^[^ ^6^, ^9^ ^]^ However, proteins are more than simply linear sequences of amino acids, and it is difficult to infer the completeness of a protein from sequence data alone. The incorporation of protein structure or functional annotation into language models is a relatively new development.^[^ ^10^, ^11^, ^12^ ^]^ Although such approaches have enhanced the performance and application of models, challenges remain. Numerous distinctive and fine‐grained protein properties, such as functional unit domains,^[^ ^13^ ^]^ have not yet been properly incorporated into a pretrained model. In addition, there is still no metric for quantifying how well a protein representation has been pretrained and how suitable it is for downstream tasks.

**Figure 1:**
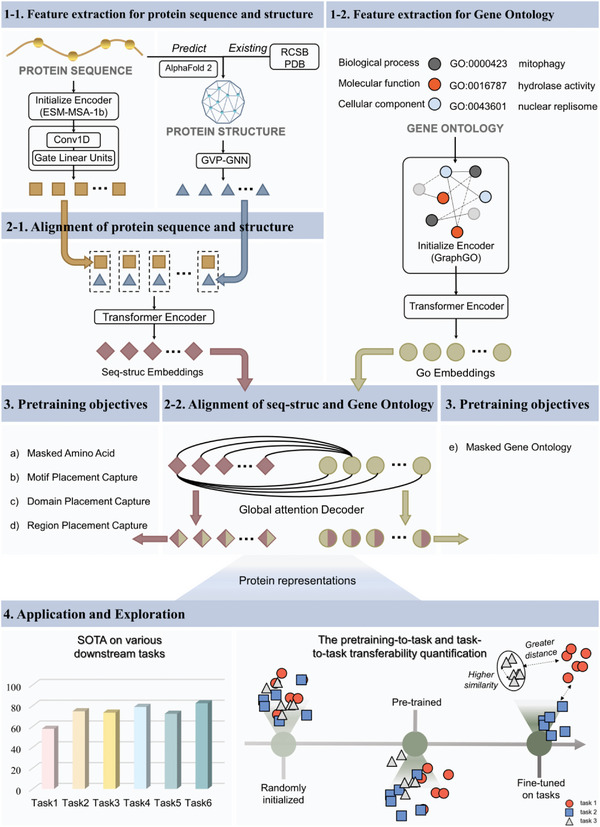
Schematic overview of the proposed framework. The framework comprises four major components. 1) Feature extraction for protein sequence, structure and Gene Ontology. 2) Protein sequence and structure alignment using token‐level self‐attention. Then, the sequence‐structure embedding is aligned globally with the GO embedding. 3) Pretraining of a multimodal model with five protein‐specific objectives. 4) Application of the resulting protein representation for downstream tasks and cross‐task learning process quantification.

Here, we introduce MASSA, a multimodal protein representation framework that incorporates domain knowledge across protein sequence, structure, and functional annotation (**Figure** **1**). The resulting protein representation is then applied for downstream tasks and cross‐task learning process quantification. It is worth noting that our model could accept sequence‐only data as input for downstream applications. When a protein sample contains three modalities, they are all taken as input, whereas for samples with missing modal (such as the protein property tasks), structure and GO (Gene Ontology) term information were processed as masked tokens.^[^ ^3^ ^]^ There are three notable improvements.

1) The rational integration of three protein modalities. The heterogeneity of protein modalities hinders their efficient fusion. Common techniques, such as concatenating the embeddings of various modalities, disregard this heterogeneity and consequently result in the loss of modality‐specific features. To solve this problem, we have developed a hierarchical two‐step alignment method. Initially, each amino acid token embedding of the protein sequence and structure is aligned using token‐level self‐attention, yielding a sequence‐structure embedding containing both sequential evolutionary and spatial information. This representation is then globally aligned with GO (Gene Ontology) annotation embedding via a cross Transformer decoder.
2) The elaborate design of pretraining objectives. Existing approaches frequently employ pretraining tasks similar to tasks from the NLP domain, such as next token prediction and masked language modeling (i.e., prediction of masked amino acids),^[^ ^6^, ^9^, ^10^ ^]^ in which physical, biochemical, and biological knowledge of proteins is not fully considered. To extract high‐level and fine‐grained protein‐domain features, we present an equalized multitask loss function for five protein‐specific pretraining objectives, including masked amino acid/Gene Ontology prediction and domain/motif/region placement capture. Based on the model architecture and comprehensive pretraining objectives, the resulting protein representation outperformed other methods in specific tasks such as protein properties (stability and fluorescence), protein–protein interaction (PPI) (shs27k/shs148k/string/skempi), and protein–ligand interaction (PLI) (kinase, DUD‐E), while achieving competitive results in secondary structure and remote homology.
3) The quantification of pretraining‐to‐task and task‐to‐task transferability. Previous studies have demonstrated that downstream tasks often benefit from pretrained models.^[^ ^6^, ^9^, ^12^ ^]^ However, this raises the question of whether such transferability can be quantified to determine how well a model has been pretrained and which downstream tasks benefit most from pretraining. Here, we introduce a novel spatial metric for quantifying the dynamic transferability from a pretrained protein representation to downstream tasks, which provides a panoramic view of the step‐by‐step learning process. With this metric, downstream task‐to‐task adaptability experiments are further explored, and a strong correlation is observed between task‐specific representation similarities and cross‐task adaptability.

## 2 Results and Discussion

### 2.1 Multimodal Data Preprocessing and Analysis

**Figure 2:**
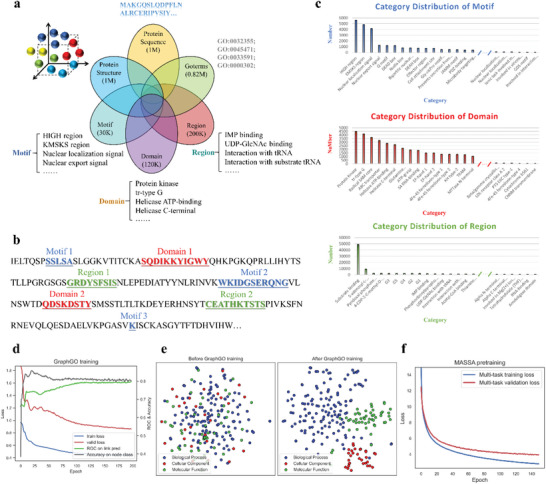
Analysis of multimodal data and model pretraining. a) Overview of the constructed multimodal dataset. b) The capture of fine‐grained protein knowledge using a method similar to named entity recognition. c) Long‐tailed distributions of motifs, domains, and regions. d) GraphGO training. e) Visualization of GO embeddings using t‐SNE before and after GraphGO training. f) The pretraining procedure of the proposed MASSA model.

We collected multiple datasets, including protein sequences from UniProt (www.uniprot.org), protein structures from RSCB PDB (www.rcsb.org) and the AlphaFold Protein Structure Database (alphafold.ebi.ac.uk), GO annotations from the Gene Ontology Resource (geneontology.org), and protein motif, region, and domain information from UniProtKB (www.uniprot.org/uniprotkb). After preprocessing, the constructed multimodal set consists of approximately one million sequence, structure, GO annotation, protein region, motif, and domain samples. The quantity and raw format of these multimodal data are depicted in **Figure** **2**a.

Among these data, the protein sequences, structures, and GO annotations were utilized as the training inputs, whereas the regions, motifs, and domains were used as the training objectives. Specifically, the term “region” refers to a region of biological interest within a sequence, such as the 346th to 375th amino acids of Q8BUZ1, which correspond to interaction with actin. A motif is a collection of secondary structures that form a particular geometric arrangement and perform a particular protein function. Because of their short lengths and high sequence variability, most motifs cannot be accurately predicted via computational methods. In contrast, a domain is an independent 3D structure folding unit that typically functions independently from the other components of a protein.

In Figure 2b, the sequence set “SSLSA” within the illustrated protein sample represents motif 1. Each amino acid in this set has its own position, knowledge, and category. To embed this information, we applied a method similar to the named entity recognition strategy used in natural language processing. Specifically, we treated each category (e.g., motif 1, domain 2) as a named entity and subdivided each entity into a combination of individual amino acids before classifying each combination. This method adheres to the BIOES notation. For instance, motif 1 is divisible into the set {*S*, *S*, *L*, *S*, *A*}, and the corresponding category set is {*B* − *Motif*1, *I* − *Motif*1, *I* − *Motif*1, *I* − *Motif*1, *E* − *Motif*1}. *B* − *Motif*1, *I* − *Motif*1 and *E* − *Motif*1 represent the “Begin”, “Inside” and “End” components, respectively, of motif 1. In addition, a nonentity amino acid (in black) is tagged as “O” (outside of entity). An entity that has only one token is tagged as “S” (single), such as *S* − *Motif*3.

Following named entity preprocessing, we analyzed the distributions of the categories. There are 1364 categories of motifs, 3383 categories of domains, and 10628 categories of regions. All of these data, as depicted in Figure 2c, have long‐tailed distributions, which tend to result in sensitivity to model training. Therefore, we merged the small subcategories in the long tail of each distribution into a single “Other” category. Finally, we obtained 130, 712, and 222 categories for motifs, domains, and regions, respectively.

### 2.2 Pretraining of a Multimodal Protein Representation

The MASSA model was then pretrained using the constructed multimodal dataset. The pretraining process consisted of three steps (Figure 1): 1) Feature extraction for each modality of sequence, structure and functional GO annotation. 2) Alignment and fusion of sequence and structure embeddings using token‐level self‐attention. Then, the resulting sequence‐structure embedding was aligned globally with the GO embedding. 3) Pretraining of the model with five protein‐specific objectives, including masked amino acid/Gene Ontology prediction and domain/motif/region placement capture.

For step 1, the initial sequence and GO embeddings were provided by the protein language model ESM‐MSA‐1b^[^ ^14^ ^]^ and GraphGO (Figure 1, a graph convolutional network for GO terms proposed in this study), respectively. The training objective of ESM‐MSA‐1b is masked language modeling, which is widely employed in the pretraining of protein language models as well as our model. As stated previously, this objective is derived from natural language processing and aims to extract protein semantics from a large number of amino acid sequences. To avoid “reinventing the wheel”, the input sequences were processed by ESM‐MSA‐1b to obtain the initial sequence embeddings for our model. It should be noted, however, that the architecture of ESM‐MSA‐1b was not connected to our model, and its model weights were not incorporated into our learning process.

GO terms are statements about diverse protein functions, which span three distinct ontology categories: biological processes, molecular functions, and cellular components (e.g., mitophagy, hydrolase activity, and nuclear replisome, respectively). To obtain the initial embedding of GO terms, we developed and trained a model called GraphGO (Figure 1). Specifically, a graph was constructed with 44733 GO terms and 150322 edges. Each GO term is represented by a node in this graph, and each edge represents a type of relationship between two GO terms (Figure S1, Supporting Information). In GraphGO, three GCN layers and two training objectives, link prediction and node classification, were employed to extract hidden features. As depicted in Figure 2d, following GraphGO training, both considered evaluation metrics, the AUC for link prediction and the accuracy of node classification, reached a high level (nearly 0.82). Moreover, the t‐SNE visualization of the GO embedding (Figure 2e) reveals excellent clustering results for the three ontologies following training. These results suggest that GraphGO has learned a reliable representation of GO terms.

MASSA was pretrained on the constructed multimodal dataset using an equalized multitask loss function for the five protein‐specific pretraining objectives. The detailed model architecture and parameters are exhibited in Extended Data Figure 1. During pretraining, the multitask loss continued to improve even after multiple epochs (Figure 2f), consistent with previous studies.^[^ ^6^, ^12^ ^]^ After 150 epochs, pretraining was stopped, and the model was then evaluated on downstream tasks.

### 2.3 Model Performance on Downstream Tasks

We evaluated and compared the pretrained MASSA model on a variety of protein‐related benchmark tasks, including protein properties, protein‐protein interactions (PPI), and protein‐ligand interactions (PLI). It is worth noting that our model doesn't necessarily require all of the sequence, structure, and function data for downstream application. When a protein sample contains three modalities, they are all taken as input, whereas for samples with missing modal (such as the protein property tasks), structure and GO term information were processed as masked tokens.^[^ ^3^ ^]^ More details can be found in Supporting Information and source code.

#### 2.3.1 Protein Property Benchmarks

We analyzed variety of protein property benchmarks from TAPE,^[^ ^9^ ^]^ including secondary structure, remote homology, fluorescence, and stability benchmarks. On these sequence‐based datasets, we evaluated the performance of our model in comparison with other methods in two distinct ways: with or without pretraining objectives. “Without pretraining objectives” (including only steps 1 and 2 in Figure 1) denotes that the model was trained from scratch on the downstream tasks, whereas “with pretraining objectives” (including steps 1–3 in Figure 1) indicates that the model was pretrained fully prior to being fine‐tuned. Both experimental groups used only protein sequence as input (i.e., structure and GO term information were masked tokens). The difference between them is that “with pretraining objectives” group could benefit from pretraining knowledge (where pretraining dataset with all three modalities were utilized).

**Figure 3:**
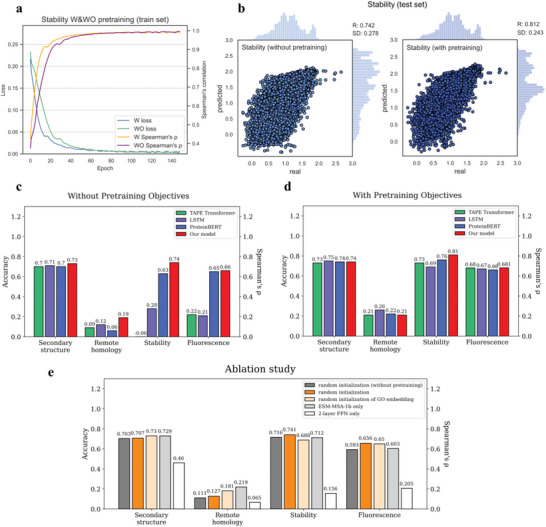
Model performance on protein property benchmarks. The evaluation metric used for the secondary structure and remote homology tasks (the left two) is accuracy, while that used for the stability and fluorescence tasks (the right two) is Spearman's *ρ*. a) Training processes of stability (with/without pretraining objectives). b) Performance on stability test set. Left: without pretraining objectives. Right: with pretraining objectives. c) Without pretraining objectives, the model was trained directly on the downstream tasks. d) With pretraining objectives, the model was pretrained before being fine‐tuned. e) Ablation study. Three methods, TAPE Transformer,^[^ ^9^ ^]^ LSTM,^[^ ^16^ ^]^ and ProteinBERT,^[^ ^12^ ^]^ are considered for comparison.

Various downstream tasks involve various types of labels. For example, the stability benchmark is a regression task in which input proteins X are mapped to continuous labels Y in order to predict protein stability. **Figure** **3**b shows that the model with pretraining achieved a Spearman's R value of 0.812 on this task, outperforming the model without pretraining (R = 0.742), indicating that pretraining greatly benefits this task. Compared to other methods, our model, as shown in Figure 3c,d, achieved state‐of‐the‐art (SOTA) performance on all tasks in the experiments without pretraining objectives. This may be explained by the fact that the “without pretraining objectives” model relies on information collected from the initial sequence (ESM‐MSA‐1b) and GO (GraphGO) embeddings. After the pretraining process, our model achieved SOTA performance on the stability and fluorescence tasks and competitive performance on the remaining tasks.

Next, we carried out an ablation study to assess the effectiveness of each module of the pretraining multi‐modal model (Figure 1). There were five experimental groups:

1) Random initialization: random vectors for the sequence and GO embedding.
2) Random initialization (without pretraining): random vectors for the sequence and GO embeddings (skip step 3 in Figure 1).
3) Random initialization of the GO embedding: without step 1–2.
4) ESM‐MSA‐1b only: the ESM‐MAS‐1b sequence embedding only (step 1‐1).
5) 2‐layer FFN (feedforward network) only: the network used to predict downstream tasks from final protein representation (i.e., without steps 1, 2, and 3, final protein representations are randomly initialized).

The presented results suggest that the initial sequence embedding from ESM‐MSA‐1b is important for these downstream tasks, especially for remote homology. Consistent with other studies,^[^ ^6^, ^14^, ^15^ ^]^ multiple sequence alignment and masked language modeling were found to be most advantageous for protein‐structure‐related tasks, such as secondary structures and remote homology. In contrast, information obtained from our proposed multimodal fusion and fine‐grained pretraining objectives is more beneficial for biophysically related tasks such as stability and fluorescence. The gaps in the results between the ablation groups and the full model (Figure 3d) collectively imply that SOTA performance can be achieved only by combining all of the modules.

#### 2.3.2 Protein‒Protein Interactions

The model was evaluated on several protein‒protein interaction benchmarks, including SHS27k, SHS148k, STRING, and SKEMPI. Among them, STRING, SHS27k, and SHS148k are multilabel classification benchmarks, while SKEMPI is built for regression. More details about these datasets can be found in the Experimental Section. Four methods, PIPR,^[^ ^17^ ^]^ GNN‐PPI,^[^ ^18^ ^]^ ProtBert,^[^ ^19^ ^]^ and OntoProtein,^[^ ^20^ ^]^ were chosen for comparison on SHS27k, SHS148k, and STRING. The models were evaluated using two heuristic schemes based on the PPI network: breadth‐first search (BFS) and depth‐first search (DFS).^[^ ^18^ ^]^ Specifically, BFS and DFS generate unknown proteins $P_{u}$ (used as the test set) in two distinct ways: 1) The $P_{u}$ interact closely with one another and are grouped as clusters within the PPI network. 2) The $P_{u}$ are widely dispersed in the PPI network and seldom interact with one another.

As seen, our multimodal model outperformed the other methods on all these benchmarks. Our model outperformed the baseline method PIPR^[^ ^17^ ^]^ and the second‐best method OntoProtein^[^ ^20^ ^]^ by an average of 41.32 and 4.74 percent, respectively.

For these PPI benchmarks, the full model receives inputs from all three modalities (sequence, structure, and GO terms), and an ablation study was conducted to assess the efficacy of each of these modalities. There were five experimental groups representing different combinations of modalities: 1) random initialization, 2) sequence only, 3) sequence + structure, 4) structure + GO, and 5) sequence + GO. As demonstrated, Groups 3–5 performed better than Group 2, suggesting that the incorporation of multiple modalities can be advantageous for all of these PPI datasets. Among these results, Groups 3 and 5, with sequence plus another modality achieved relatively superior outcomes, confirming the significance of sequence information.

**Figure 4:**
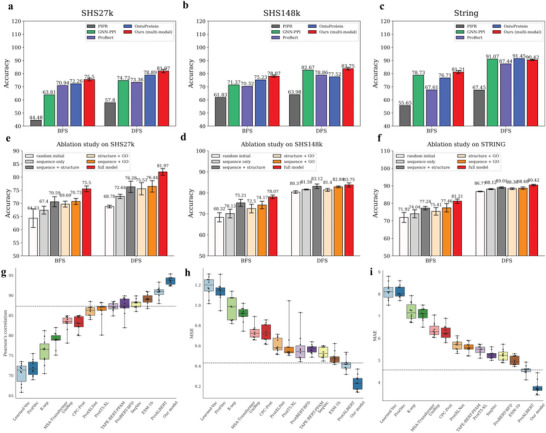
Model performance on protein‒protein interaction benchmarks. a–c) SHS27k, SHS148k, and STRING. Four methods, PIPR,^[^ ^17^ ^]^ GNN‐PPI,^[^ ^18^ ^]^ ProtBert,^[^ ^19^ ^]^ and OntoProtein,^[^ ^20^ ^]^ are chosen for comparison. e,f) Ablation study on SHS27k, SHS148k, and STRING. Breadth‐first search (BFS) and depth‐first search (DFS) are the proposed schemes for splitting the datasets. The mean and standard deviation values for five experimental runs are reported. g–i) Performance comparisons of 13 methods^[^ ^21^ ^]^ on the SKEMPI dataset. g) Pearson's correlation coefficients reflecting the correlation between the predicted and real binding affinities (higher is better) h) Mean squared error values, multiplied by 100 (lower is better). i) Mean absolute error values, multiplied by 100 (lower is better). The best result from PIPR is indicated by a dotted black line. Each dot represents the performance for one fold in the 10‐fold cross‐validation.

Many previous prediction models have been applied to SKEMPI, which is a regression dataset for predicting the influence of mutation‐based (i.e., with one or more amino acids replaced) changes in binding affinity on PPI. Our model was evaluated on this dataset using 10‐fold cross‐validation and three metrics, namely, Pearson's correlation (higher is better), mean squared error (MSE) (lower is better), and mean absolute error (MAE) (lower is better). The 10‐fold training process for the SKEMPI set is shown in detail in Figure S2 (Supporting Information). We then compared our model to the baseline method PIPR^[^ ^17^ ^]^ and 13 other protein language models.^[^ ^21^ ^]^ As shown in **Figure** **4**g,h, our multimodal model produced SOTA results, with MSE = 0.23, MAE = 3.78, and Pearson's correlation = 93.1%, outperforming the baseline method PIPR^[^ ^17^ ^]^ (MSE = 0.63, MAE = 5.48, and Pearson's correlation = 87.3%) and the second‐best method ProtALBERT^[^ ^21^ ^]^ (MSE = 0.43, MAE = 4.57, and Pearson's correlation = 90.7%). These results suggest that the additional information provided by the rational integration of multiple modalities may be advantageous for PPI tasks.

#### 2.3.3 Protein‒Ligand Interactions

We also evaluated our model on protein‒ligand interaction (PLI) tasks. Two PLI benchmarks were used: the Kinase set (sequence‐based), PDBbind v.2016 (structure‐based), and DUD‐E (structure‐based). Similar to the PPI experiments described previously, our model received inputs from all three modalities. The structures of proteins from the Kinase set used in this evaluation were predicted by Alphafold2. Another point to note is the PLI architecture; ligand embedding was provided by Attentive‐FP^[^ ^22^ ^]^ (a graph‐attention‐network‐based method), and protein‒ligand interactions were processed by a 3‐layer FFN.

We split PDBbind into training/validation/test sets similarly to what was done for Pafnucy (a classical structure‐based deep learning method) and compared the results using two metrics: Pearson's correlation coefficient R and root mean square error (RMSE). As shown in Extended Data Figure 2, our model outperformed Pafnucy (RMSE = 1.42 and R = 0.78) and obtained competitive results (RMSE = 1.327 and R = 0.794) compared to those of the SOTA models, which attained results similar to RMSE = 1.28 and R = 0.82. It is worth noting that most methods compared on the PDBbind set aim to predict the binding affinity based on the structure of the protein‒ligand complex, whereas in our model, the proteins and ligands are input separately and the actual binding mode is unknown. On the other benchmark, the Kinase set, our model achieved SOTA performance (AUC = 0.737, PRC = 0.454) (Extended Data Figure 2), outperforming the previous SOTA method TransformerCPI^[^ ^23^ ^]^ (AUC = 0.598 and PRC = 0.280). For DUD‐E, the target‐clustered split technique is applied to avoid protein redundancy between the train and test sets. Our model is tested using 3‐fold cross validation, and proteins are grouped using global sequence alignment to ensure that targets with sequence identities exceeding 70% are included in the same fold during cross validation. The AUC results indicate that our model performs well on DUD‐E dataset, with a mean AUC of 0.972 for target clustering with 70% sequence identity.

#### 2.3.4 Brief Summary

The results mentioned above can be summarized as follows.

1) Our model achieved SOTA results on most protein‐related benchmarks, particularly protein‒protein interactions (nearly 5% superior to the second‐best method). The PPI task is designed to discover the relationship between two proteins. A multimodal model can inject more domain‐specific information into the protein representation, thus enhancing the ability to discover such relationships. Specifically, the GO terms can provide information about protein functions, such as biological processes and cellular locations. When two proteins are found in the same cellular component and are involved in the same biological process, the likelihood of their interaction increases, and vice versa.
2) The efficacy of each module was revealed. The effectiveness of the initial sequence and GO embeddings as well as various combinations of modalities was investigated in two ablation studies. The presented results indicate that the initial sequence embedding is especially useful for protein‐structure‐related tasks, such as secondary structures and remote homology, whereas biophysically related tasks, such as stability and fluorescence, benefit more from domain knowledge introduced through multimodal pretraining. For multiple modalities, the results suggest that combinations of sequence data with another modality (structure or GO) produce relatively superior results.
3) Properly incorporating multiple modalities is important. Based on the findings, we conclude that the model can have the best effect when all multimodal data, including sequence, structure, and GO information, are provided as input. For instance, when only sequence information was used as input for the four protein property benchmarks while the structure and GO inputs to our model were masked, it was obvious that the model's performance was severely hampered by the lack of other modalities, as further evidenced by the PPI benchmark results. When all available multimodal data were used, our model outperformed other SOTA approaches on almost all PPI benchmarks. In the ablation study, model variants using fewer modalities exhibited worse performance under the same circumstances, further validating our conclusion. On the other hand, the PLI task may depend on the ligand representation and the protein‒ligand interaction module in addition to the protein representation. It is possible that a more complex interaction module (e.g., in which the potential binding pocket can be set) would improve the model performance on the PLI task.

### 2.4 Ablation Study on Homologous Proteins

In order to make fair comparisons with other landmark methods, the data partitioning of all the applied downstream tasks were obtained from their original sources (more details can be found in Experimental Section and Supporting Information). However, the protein homologous issues within these benchmarks merit discussion. The presence of homologous proteins in both the training and test sets can easily lead to overfitting. Consequently, we examined the protein homology of each of these benchmarks using MMseqs2^[^ ^24^ ^]^ and conducted protein sequence homology ablation experiments with 25% sequence identity threshold.

Briefly, we removed proteins from the train set whose sequence identity with the other sets exceeded 25%, and then we trained and evaluated the performance of the model. On these homology removal datasets, we then run our model MASSA and other methods (using their released codes and model weights on GitHub). As shown in Supporting Information, our model outperforms other methods on these homology removal datasets. Notably, all deep learning models have demonstrated significantly better performance than models with few parameters (i.e., SVM and Random Forest). These results indicate that our model was not overtrained and has superior predictive ability compared to other state‐of‐the‐art methods in the presence of few homologous training samples.

### 2.5 Transferability of the Feature Space Representation Across Tasks

The above results verify the effectiveness of transfer learning from a pretrained multimodal representation to downstream tasks, consistent with previous studies.^[^ ^1^, ^6^, ^9^ ^]^ However, there are still many challenges that need to be further addressed in this field. First, is it possible to quantify such transferability, thereby determining how well a model has been pretrained and the extent to which downstream tasks benefit from pretraining? Another interesting issue is the adaptability across downstream tasks. Specifically, what is the relationship between the distributions of the intertask feature spaces and their mutual transferability?

Here, to measure the dynamic transferability of a multimodal representation to downstream tasks, we introduce a novel optimal‐transport‐based metric for quantifying the cross‐task learning process (more details can be found in the Experimental Section).

#### 2.5.1 The Dynamic Transferability of a Pretrained Model to Downstream Tasks

We expect that in a well‐formed feature space, there should be a high level of intratask distributional similarity and a low level of intertask distributional similarity. To quantify the dynamic transferability, we introduce a novel metric called the optimal‐transport‐based feature space representation measure (OTFRM). This metric is intuitively the ratio of the intratask similarity (i.e., the similarity of the feature space distribution within a given task) to the average intertask similarity between the given task and other tasks (more details can be found in the Experimental Section). This value makes intuitive sense because a larger value indicates a closer spatial distribution for the given task and a greater spatial separation between the given task and other tasks.

**Figure 5:**
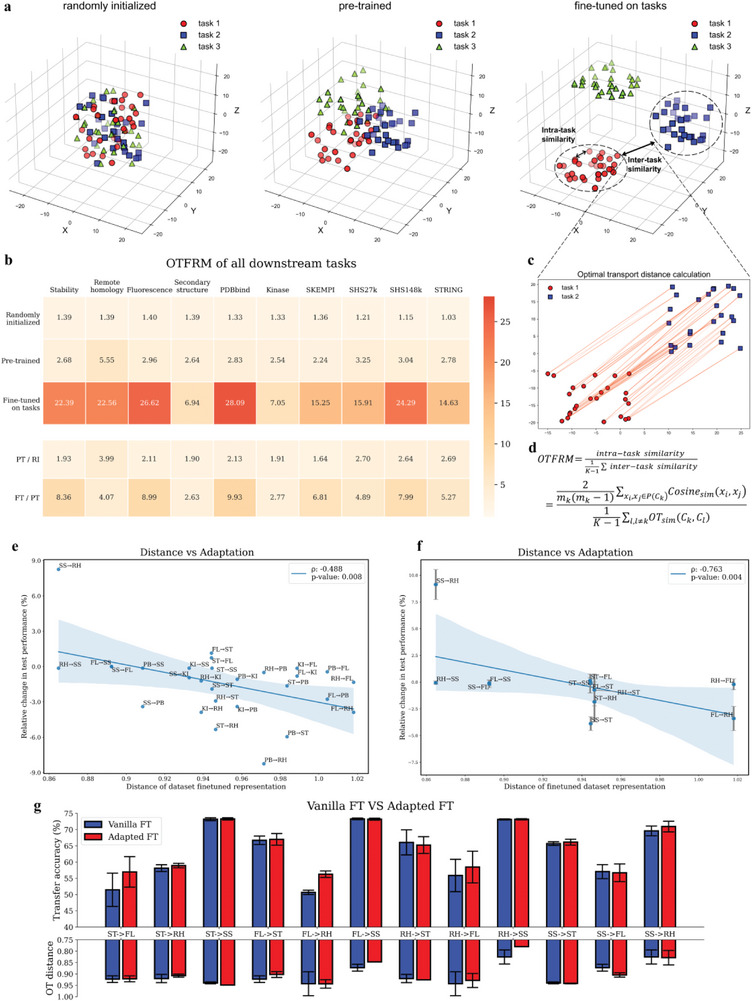
Analysis of the dynamic transferability of the cross‐task learning process. a) The distributions of tasks in the multimodal feature space at different stages: randomly initialized, pretrained, and fine‐tuned on the tasks. b) The OTFRM scores of all downstream tasks. “Randomly initialized”, “pretrained” and “fine‐tuned” refer to the stages of random initialization (before pretraining), multimodal pretraining, and fine‐tuning on downstream tasks. PT/RI and FT/PT denote the ratios of the pretrained results to the randomly initialized results and of the fine‐tuned results to the pretrained results, respectively. c) The calculation of intertask similarity by means of optimal transport. d) The proposed OTFRM formula. e,f) The relationship between the intertask distribution distances and the mutual adaptability between the corresponding tasks. f) The results of adaptation experiments performed five times with distinct random seeds for all protein property benchmarks. The farther apart the distributions of two datasets are, the lower their mutual adaptability. g) Comparison of empirical results and OT distances using Vanilla‐FT and Adapted‐FT (OTFRM‐guided fine‐tuning). Abbreviations: ST (stability), FL (fluorescence), SS (secondary structure), RH (remote homology), PB (PDBbind), KI (Kinase). The mean and standard deviation values for five experimental runs are reported.

As depicted in **Figure** **5**, the feature distributions for all tasks without pretraining (randomly initialized representations) are mixed, as evidenced by their similar small OTFRM values. The increase in the OTFRM values following pretraining indicates that all of these tasks benefit from pretraining. Then, as expected, after fine‐tuning, all values are significantly increased. In addition, the ratios of the with‐pretraining to without‐pretraining values and the fine‐tuned to with‐pretraining values were calculated. Obviously, the degrees of transferability from the pretrained model to various downstream tasks vary. For example, remote homology benefits more than other tasks from pretraining. This observation is consistent with the experimental finding that the model can easily achieve high performance on remote homology within the first few epochs of fine‐tuning. These dynamic alterations of the feature space distributions across tasks validate our hypothesis, revealing how much these tasks benefit from each learning step.

#### 2.5.2 Adaptability Across Downstream Tasks

Based on the aforementioned findings, we hypothesized that the heterogeneity of the feature space distributions of tasks might affect how well the corresponding feature representations can be transferred between tasks. Accordingly, we investigated the adaptability across downstream tasks. Using the intertask distribution difference operator from the OTFRM, we first calculated the pairwise distances between the spatial distributions of different downstream tasks based on the feature space of the multimodal representation. Among these downstream tasks, we then conducted a number of adaptation experiments. For instance, we trained the refined model for task A on task B until the models converged (e.g., A→B, stability→fluorescence). To determine whether the adaptive performance was improving or degrading, it was compared to the initial performance for task A. The relationship between the intertask distribution distances and the mutual adaptability of the tasks was then calculated.

The pairwise distances between the tasks gradually increased as the phases progressed, consistent with the above results (Figure S3, Supporting Information). The results shown in Figure 5e,f suggest that the metric is highly predictive of the adaptability across downstream tasks. According to Figure 5g, there is a significant correlation between the intertask feature space distributions and adaptability (Spearman's *ρ* = −0.488). In other words, the adaptation performance is more likely to decrease when tasks A and B are farther apart. For instance, there was a decline in performance of more than 6 points in the adaptation from PDBbind to stability. In addition to the protein representation, the PLI and PPI tasks may also rely on the interaction module, which may introduce additional noise. Therefore, an attempt was made to conduct experiments solely on protein property benchmarks. We performed the adaptation tasks five times with distinct random seeds for each pair of protein property benchmarks and computed the confidence intervals. As demonstrated, the Spearman correlation rose to −0.763. Collectively, these findings corroborate the utility of the OTFRM for elucidating dynamic transferability and suggest that it can be used to predict adaptability across downstream tasks as well.

#### 2.5.3 OTFRM‐Guided Fine‐Tuning can Improve Model Performance

Considering that the OTFRM can accurately measure the similarities across downstream tasks, we designed an OTFRM‐guided fine‐tuning strategy named Adapted‐FT. Specifically, unlike the traditional fine‐tuning method (Vanilla‐FT), in which a model for the target task B is directly fine‐tuned based on a model for the source task A, Adapted‐FT consists of three steps (as depicted in Extended Data Figure 3). Initially, the pretrained model *θ*_PT_ is trained on source task A. The model is then alternately optimized on source task A and target task B to maximize transferability using the OTFRM. Finally, the model is fine‐tuned on target task B. The proposed Adapted‐FT strategy was then applied on the four protein property benchmarks five times with different random seeds. As seen in Figure 5g, Adapted‐FT outperformed Vanilla‐FT in terms of both transfer accuracy and OT distance for many tasks (e.g., for ST→FL and FL→RH, 16% and 10% improvements, respectively, in transfer accuracy were achieved), demonstrating that the OTFRM can effectively direct the adaptation across tasks. This method can be used to further enhance model performance on downstream tasks.

## 3 Conclusion

In recent years, we have witnessed the emergence of an increased number of AI‐based computational approaches for learning protein representations, which are essential in downstream biological applications. Most existing methods are usually trained using only a single data format, such as protein sequences. However, protein knowledge can be accumulated from numerous types of biological experiments. In this study, we have proposed a multimodal protein representation framework for incorporating domain knowledge across protein sequence, structure, and function information. With careful design of the pretraining process, we were able to create a protein representation learning tool with broad applicability (github.com/SIAT‐code/MASSA). We have demonstrated that our model performs exceptionally well on all protein‐related tasks, exceeding state‐of‐the‐art levels, particularly when all multimodal data are used as inputs. Specifically, we argue that for effective protein representation learning, three critical factors should be considered, as follows:

1) Appropriate data type selection and preprocessing. The majority of protein representation methods are based on sequence data due to the massive available corpora. However, as a result of DeepMind's outstanding efforts, a wealth of information on protein structures is also available.^[^ ^15^ ^]^ Therefore, a multimodal protein model will have broader applicability. We showed that incorporating multiple protein modalities can result in better performance on a variety of downstream tasks. However, combining different modalities of protein‐related data is difficult due to the complex characteristics of these data, such as high‐dimensional sparse features. We extracted knowledge from domain/motif/region information using a named entity recognition strategy. For other types of protein data, however, such as post‐translational modification, additional techniques will be needed.
2) Elaborate model architecture. The alignment and fusion of different modalities are critical in multimodal machine learning.^[^ ^25^ ^]^ The heterogeneity of information across modalities may impede efficient fusion. Each protein modality is associated with a private subspace that captures domain‐specific features and a shared subspace that captures shared features.^[^ ^26^ ^]^ Performing multimodal fusion to early may result in the loss of modality‐specific features. Therefore, we extracted the features of each protein modality separately, including evolutionary and spatial information for sequence and structure data, respectively. Then, based on the characteristics of the different modalities, they were aligned at either the token or global level. This technique can preserve more modality‐specific features than conventional methods, such as concatenating the modal embeddings in a late stage without alignment. Under the assumption that there are both domain‐specific and shared features across modalities, more intriguing methods could be attempted. For instance, a better representation might be obtained by training a domain classifier to minimize domain‐shared features and maximize domain‐specific features.^[^ ^27^ ^]^
3) Suitable training objectives. The relevant training objectives, which guide model optimization and feature extraction, should be given proper priority in representation learning. However, when defining pretraining objectives, existing approaches have relied significantly on experience obtained from natural language processing (e.g., masked language modeling). We have introduced more protein‐specific training objectives to extract high‐level and fine‐grained protein‐domain features. Although their effectiveness has been confirmed, there is still considerable room for improvement. For instance, it is still unclear which features obtained based on which objectives contribute most to a particular biological task. The rational design of a series of training objectives to encapsulate the desired properties for a given task remains a challenge.
4) Future research direction: rationally designing protein representation learning by quantifying transferability. It should be highlighted that different biological properties of a protein will place varying demands on the representations for various downstream tasks, and it is difficult to embed all desired properties in one representation. Therefore, it appears that using a single protein representation for all tasks, while appealing, is not feasible in reality. The relationship between biological properties and hidden features, as well as which components should be included in the pretraining phase in order to capture and transfer the most desirable biological properties for the given task, should be quantitatively analyzed. Here, we have presented a novel metric for quantifying the dynamic transferability of a pretrained protein representation to downstream tasks. We have shown that this metric can be utilized to evaluate the cross‐task learning process, predict adaptability and guide fine‐tuning across various tasks. Future work will focus on improving the transferability metric to guide the design of neural networks and training objectives for protein representation learning.

## 4 Experimental Section

### Data

*Multimodal Pretraining Data*: A multimodal protein dataset was constructed for pretraining the protein representation. Three types of data were used: sequence, structure, and functional annotation data.

Sequences and GO annotations from UniProtKB Swiss‐Prot (https://www.uniprot.org/) were used to compile the multimodal protein dataset. For 3D structure embedding, protein structures from the AlphaFold Protein Structure Database (AlphaFold DB: https://alphafold.ebi.ac.uk/download) and the RCSB PDB (https://www.rcsb.org/) were used. The structures predicted by AlphaFold2 may not be accurate. This had been examined in the Supporting Information. To obtain fine‐grained domain knowledge, protein data at the amino acid level, such as regions, motifs and domains, were extracted from UniProtKB as training objectives. Finally, the constructed multimodal protein dataset consists of nearly one million samples, each of which contains sequence and structure data and many of which contain GO annotations, regions, motifs, and domains (Figure 2a).

To achieve an efficient preliminary representation and to save training time, two initial encoders were utilized for the sequences and GO annotations before multimodal pretraining. Specifically, for the initial sequence encoder, ESM‐MAS‐1b was trained on a dataset of 26 million MSAs, in which the average depth was 1192. An MSA was generated for each UniRef50 sequence by searching UniClust30 with HHblits (detailed in^[^ ^14^ ^]^). For the initial GO annotation encoder, GO terms and their relationships from the Gene Ontology Resource were used to build a GO graph containing 44733 nodes and 150322 edges.

To evaluate the pretrained multimodal model, It was further tested on three protein‐related tasks.

*Protein Property Benchmarks*: Four protein property benchmarks covering stability, fluorescence, remote homology, and secondary structure were used. Among them, three datasets (stability, fluorescence, remote homology, and secondary structure) were taken from TAPE,^[^ ^9^ ^]^ which was a set of semisupervised biological benchmarks for evaluating the performance of protein language models.

*Protein‒Protein Interaction Datasets*: Four protein‒protein interaction datasets with three types of tasks were used in this study.

The Structural Kinetic and Energetic database of Mutant Protein Interactions (SKEMPI)^[^ ^28^ ^]^ was used for a regression task, which was constructed to investigate the influence of mutation‐based binding affinity changes on PPI. SKEMPI contains 3047 binding affinity changes based on amino acid substitutions within protein complexes. The unit of binding affinity is the equilibrium dissociation constant (K_d_). The lower the K_d_ value is, the better the binding affinity. Chen et al.^[^ ^17^ ^]^ further averaged the Kd values of duplicate entries to obtain 2950 PPI samples, which had served as a comparative benchmark for many methods.^[^ ^21^ ^]^

The STRING database integrates the majority of publicly accessible protein‒protein interaction data to construct a global PPI network that includes both direct (physical) and indirect (functional) interactions. Here, the classification of PPI was focused by STRING for multiple types. This database categorizes PPI into the following seven types: reaction, binding, posttranslational modification (ptmod), activation, inhibition, catalysis, and expression. At least one of them was present in every pair of interacting proteins. In a previous study 1690 and 5189 proteins with 40% sequence identity were selected from the Homo sapiens subset of STRING to generate SHS27k and SHS148k, which contain 7624 and 44488 multilabel PPI, respectively. Simultaneously, all PPI of Homo sapiens were used as the third dataset, STRING, containing 15335 proteins and 593397 PPI.^[^ ^18^ ^]^

*Protein‒Ligand Interaction Datasets*: Three protein‒ligand interaction datasets were used here, PDBbind,the Kinase set, and Directory of Useful Decoys Enhanced (DUD‐E). The PDBbind v2016^[^ ^29^ ^]^ core set, with 290 diverse complexes covering all protein classes in the PDBbind refined set, was used as a test set for comparisons with other methods. The PDBbind dataset split was the same as in our previous study.^[^ ^30^ ^]^ First, the core set containing 290 protein‒ligand complexes was utilized as the test set. Second, 1000 complexes in the refined set were separated to be used as a validation set (same as Pafnucy^[^ ^31^ ^]^). Third, the other complexes from the refined and general sets were combined for use as the training set. Thus, a total of 13196 complexes were used for model evaluation. The Kinase dataset was taken from TransformerCPI,^[^ ^23^ ^]^ which was derived from the KIBA set,^[^ ^32^ ^]^ containing 229 proteins, 1644 ligands, and 111237 interactions. The DUDE dataset includes 102 targets belonging to eight broad protein categories, such as kinases, proteases, nuclear receptors, etc., as well as 22886 active compounds and their affinities against 102 targets, with an average of 224 ligands per target.^[^ ^33^ ^]^

### Multimodal Representation Model Architecture

The model construction and training process consisted of three main steps. First, the input protein sequences, structures, and GO annotations were processed by ESM‐MSA‐1b,^[^ ^14^ ^]^ GVP‐GNN,^[^ ^34^ ^]^ and GraphGO (Figure 1b) modules, respectively. More specifically, ESM‐MSA‐1b was pretrained and taken from MSA Transformer,^[^ ^14^ ^]^ which takes an MSA matrix as input and outputs the probabilities of masked amino acids. The GVP‐GNN module,^[^ ^34^ ^]^ which could perform both geometric and relational reasoning on the 3D structures of proteins, was used to obtain the initial 3D structure embedding without pretraining. The architecture of GraphGO, which takes a GO annotation network as input and could output the embedding of each GO node, is depicted in Figure 1b. GraphGO was designed here to learn robust representations of GO nodes and their relationships using multitask learning for link prediction and node classification. It should be noted that ESM‐MSA‐1b and GraphGO were only used to obtain the initial embeddings, and their network weights were not updated in subsequent epochs of multimodal pretraining.

Second, after preprocessing, the representations of protein sequence and structure were aligned at the token level using a Transformer encoder. This representation was then globally aligned with the GO annotation representation through a cross Transformer decoder. Third, the output multimodal representations of the protein and GO features were trained on several protein‐specific tasks, including predictions of masked amino acids and GO terms and predictions of amino acids and locations within protein regions, motifs and domains. The loss functions are described in detail as follows.

### Structural Encoder (GVP‐GNN)

GVP‐GNN module^[^ ^34^ ^]^ was used to process raw protein structure (typically .pdb). It consists of two inputs, scalar feature and geometric feature, which represent the attributes and 3D coordinates of amino acids, respectively. Scalar features provide rotational invariance of molecules, whereas geometric features provide an absolute direction for each amino acid node, as opposed to the relative direction between neighbors, making it easier for GNN to access the global geometric characteristics of the structure. Using GVP instead of multi‐layer perceptron (MLP) could preserve rotation invariance and global geometric properties during the graph propagation process. More details can be found in Supporting Information.

### Loss Function

The loss function minimized by the model during pretraining was a sum of a categorical cross‐entropy loss and an equalized focal loss,^[^ ^35^ ^]^ which was designed to address the classification task for long‐tailed distributions. Typically, the multiclass cross‐entropy (CE) loss (where the label is 0 or 1) is defined as:

(1) $$CE\left(p,q\right)=-\sum_{i=1}^{C}p_{i}\log\left(q_{i}\right)$$

where the number of classes is C and *p* and *q* correspond to real and predicted distributions, respectively. Then, the equalized focal loss (EFL) was applied for multiclass classification with long‐tailed distributions. To address the issue of data imbalance, a focusing factor was added to the CE expression; thus, the EFL is defined as:

(2) $$EFL\;\left(p_{t}\right)=-\alpha_{t}{\left(1-p_{t}\right)}^{\gamma^{j}}\log\left(p_{t}\right)$$

where *p*_t_ ∈ [0, 1] represents the confidence score prediction for a given target and *α*_t_ is a weighting factor for balancing positive and negative samples. Then, the focusing factor *γ* ^*j*^ is further decomposed into two factors: $\gamma^{j}=\gamma_{b}+\gamma_{v}^{j}=\gamma_{b}+s\left(1-g^{j}\right)$, where *γ*_*b*_ is a focusing factor that controls the basic behavior of the classifier in the balanced data scenario and $\gamma_{v}^{j}\geq 0$ is a variable factor associated with the level of imbalance in the *j*‐th category. In addition, another set of weights is added to rebalance the loss contributions of different categories, where high weighting factor values are assigned to increase the loss contributions of rare categories while maintaining the weighting factors for common categories near 1. The weighting factor of the *j*‐th category is represented as $\frac{\gamma_{b}+\gamma_{v}^{j}}{\gamma_{b}}$. Finally, the EFL is defined as:

(3) $$\mathrm{EFL}\;\left(p_{t}\right)=-\sum_{j\;=\;1}^{C}\alpha_{t}\left(\frac{\gamma_{b}+\gamma_{v}^{j}}{\gamma_{b}}\right){\left(1-p_{t}\right)}^{\gamma_{b}+\gamma_{v}^{j}}\log\left(p_{t}\right)$$

### Optimal‐Transport‐based Feature Space Representation Measure

Here, a novel metric, namely, the optimal‐transport‐based feature space representation measure (OTFRM) was proposed, to measure the dynamic transferability from a multimodal representation to downstream tasks.

*Background on Optimal Transport*: Optimal transport (OT) theory was first derived from the Monge problem, and later, the Kantorovich relaxed OT problem was proposed to find a transport plan describing how to move some measure to another measure of the same mass such that a certain cost function is minimized.^[^ ^36^ ^]^ OT theory was a powerful and principled method for comparing probability distributions due to its inherent properties.^[^ ^37^ ^]^ For a complete and separable metric space X, along with continuous or discrete probability measures α∈P(X) and β∈P(X), the Kantorovich OT problem is defined as:

(4) OTα,βΔ=minπ∈Πα,β∫X×Xcx,ydπx,y

where $c\left(.,\;.\right):X\times X\to R^{+}$ is a cost function and Π(*α*, *β*) is a set of couplings consisting of joint distributions over the product space X×X with marginal distributions *α* and *β*:

(5) Πα,βΔ={π∈PX×X|P1#π=α,P2#π=β}

Consider a metric $d_{X}$; if the ground‐truth cost satisfies $c\;\left(x,\;y\right)=d_{X}\;{\left(x,y\right)}^{p}$ for some *p* ≥ 1, then it have the *p*‐Wasserstein distance:

(6) Wpα,βΔ=OTα,β1/p

Since obtaining the true measures *α* and *β* is almost infeasible in practice, discrete empirical measures $\alpha =\sum_{i=1}^{m}a_{i}\delta_{x^{i}}$ and $\beta =\sum_{i=1}^{n}b_{i}\delta_{y^{j}}$ are usually used instead, where **a** and **b** are vectors in the probability simplex. In this case, the pairwise costs in Equation (4) can be compactly represented as an *m* × *n* matrix *C*, where *C_ij_* = *c*(x^*i*^, y^*j*^).

With the addition of an entropy‐regularized term, the OT problem defined in Equation (4) becomes the entropy‐regularized problem, which could then be efficiently solved by using the Sinkhorn algorithm.^[^ ^38^, ^39^ ^]^

*The OTFRM*: Intuitively, a well‐formed feature space should have high intraclass similarity and low interclass similarity. Here, it was assumed that the cross‐task feature space possesses comparable qualities. Consequently, the OTFRM was proposed for evaluating the quality of feature representations by quantifying the level of intratask distributional similarity versus intertask distributional similarity based on the multimodal representation feature space.

The cosine similarity was employed as the fundamental distance metric based on two considerations: 1) A high‐dimensional feature space necessitates a similarity that performs well in high dimensions. 2) The cosine similarity satisfies *Cosine_sim_* = 1 − *Cosine_dist_*, allowing to define similarity based on the corresponding distance. For the feature probability distributions *α* and *β* of two tasks *C*_k_ and *C*_l_, using the cosine distance as the cost metric $d_{X}$ in the equation for the *p*‐Wasserstein distance, the OT similarity of the two tasks can be defined as:

(7) $$\begin{array}{c} OT_{sim}\left(C_{k},\;C_{l}\right)=1-W_{p}\;\left(\alpha ,\beta \right)=1-OT{\left(\alpha ,\beta \right)}^{1/p}\\ \;=1-{\left[\min_{\pi \in \Pi \left(\alpha ,\beta \right)}\sum_{i,\;j\;=\;1}^{m,\;n}Cosine_{dist}{\left(x_{i},\;y_{j}\right)}^{p}\pi_{ij}+\varepsilon H\left(\pi \right)\right]}^{\frac{1}{p}} \end{array}$$

where *m* and *n* are the numbers of samples in *C*_k_ and *C*_l_, respectively; *π* is a coupling matrix of size *m* × *n*; and $H\;\left(\pi \right)=-\sum_{i\;=\;1}^{m}\sum_{j\;=\;1}^{n}\pi_{ij}\log\pi_{ij}$ is an entropy‐regularized term with *ε* = 0.1.

For a feature space containing *K* tasks, where *C*_k_ is the inspected task, the intertask similarity could be defined as the sum of the OT similarities of all unique pairs between samples in *C*_k_ and samples outside of *C*_k_, normalized by the number of unique pairs:

(8) $$inte\;r_{sim}=\frac{1}{K-1}\sum_{l,\;l\neq k}OT_{sim}\left(C_{k},\;C_{l}\right)$$

Considering that the intratask similarity should share the same scale as the intertask similarity, the intratask similarity was defined as the mean cosine similarity of all unique combinations within the inspected task:

(9) $$intr\;a_{sim}=\frac{2}{m_{k}\left(m_{k}-1\right)}\;\sum_{x_{i},x_{j}\in P\left(C_{k}\right)}Cosine_{sim}\left(x_{i},\;x_{j}\right)$$

Then, the OTFRM is defined as:

(10) $$OTFRM=\frac{\frac{2}{m_{k}\left(m_{k}-1\right)}\sum_{x_{i},x_{j}\in P\left(C_{k}\right)}Cosine_{sim}\left(x_{i},\;x_{j}\right)}{\frac{1}{K-1}\sum_{l,\;l\neq k}OT_{sim}\left(C_{k},\;C_{l}\right)}$$

Both the intra‐ and intertask similarity fall within [−1, 1]. The greater the score is, the closer the spatial distribution of the given task and the greater the separation between the given task and other tasks. By inspecting each task with support from the other tasks, a specific measure was quantitatively obtained for cross‐task feature space representation.

### Evaluation Metrics

For regression tasks, Pearson's correlation coefficient R and the root mean square error (RMSE), which reflect the linear correlation and the difference between the predicted and real values, respectively, were used as evaluation metrics:

(11) $$RMSE=\sqrt{\frac{1}{N}\sum_{i=1}^{N}{\left(y_{i}-{\hat{y}}_{i}\right)}^{2}}$$

(12) $$R=\frac{\sum_{i=1}^{N}\left(y_{i}-\bar{y}\right)\left({\hat{y}}_{i}-\bar{\hat{y}}\right)}{\sqrt{\sum_{i=1}^{N}{\left(y_{i}-\bar{y}\right)}^{2}}\sqrt{\frac{1}{N}\sum_{i=1}^{N}{\left({\hat{y}}_{i}-\bar{\hat{y}}\right)}^{2}}}$$

where N is the size of the dataset, *y*_i_ is the *i*‐th real value, *ŷ*_i_ is the *i*‐th predicted value, $\bar{\hat{y}}$ is the average of the real values, and $\bar{y}$ is the average of the predicted values.

For classification tasks, several metrics, including accuracy, precision, recall and the Matthews correlation coefficient (Mcc), were used to evaluate the model performance on an imbalanced dataset:

(13) $$Accuracy=\frac{TP+TN}{P+N}$$

(14) $$Precision=\frac{TP}{TP+FP}$$

(15) $$Recall=\frac{TP}{TP+FN}$$

(16) $$Mcc=\frac{TP\times TN-FP\times FN}{\sqrt{\left(TP+FP\right)\left(TP+FN\right)\left(TN+FP\right)\left(TN+FN\right)}}$$

where TP, TN, FP, and FN indicate the numbers of true positives, true negatives, false positives, and false negatives, respectively. Here, P and N indicate positive and negative, respectively.

*Statistical Analysis*: Results were presented as the mean ± SD. The Independent Sample t‐test was used to determine the statistical significance. All evaluation metrics were computed using the sklearn and scipy Python libraries.

## Conflict of Interest

The authors declare no conflict of interest.

## Author Contributions

F.H., Y.H., and W.Z. contributed equally to this work. F.H. and P.Y. led the research. F.H. and P.Y. contributed technical ideas. F.H., Y.H, and W.Z. developed the proposed method. F.H., Y.H., W.Z., and H.H. performed analysis and experiments. Y.P. provided evaluation and suggestions. All authors contributed to the manuscript.

## Supporting information

Supporting Information

## Data Availability

The data that support the findings of this study are openly available in Github at https://github.com/SIAT‐code/MASSA, reference number 9.
