# Supplementary material for: A Multimodal Protein Representation Framework for Quantifying Transferability Across Biochemical Downstream Tasks
Source: Adv Sci (Weinh). 2023 May 30;10(22):2301223. doi: 10.1002/advs.202301223 (PMC10401162; doi:10.1002/advs.202301223)
Supplement: Supplementary file 1 — Supporting Information [file ADVS-10-2301223-s001.pdf]

ADVANCED SCIENCE
Open Access

Supporting Information

for *Adv. Sci.*, DOI 10.1002/advs.202301223

A Multimodal Protein Representation Framework for Quantifying Transferability Across Biochemical Downstream Tasks

*Fan Hu, Yishen Hu, Weihong Zhang, Huazhen Huang, Yi Pan and Peng Yin**

Supplementary Information for

A Multimodal Protein Representation Framework for Quantifying Transferability Across Biochemical Downstream Tasks

Fan Hu[#], Yishen Hu[#], Weihong Zhang[#], Huazhen Huang, Yi Pan and Peng Yin[*]

Guangdong-Hong Kong-Macao Joint Laboratory of Human-Machine Intelligence-Synergy Systems, Shenzhen Institute of Advanced Technology, Chinese Academy of Sciences, Shenzhen, 518055, China

#: These authors contributed equally to this work.

*To whom correspondence should be addressed: peng.yin@siat.ac.cn

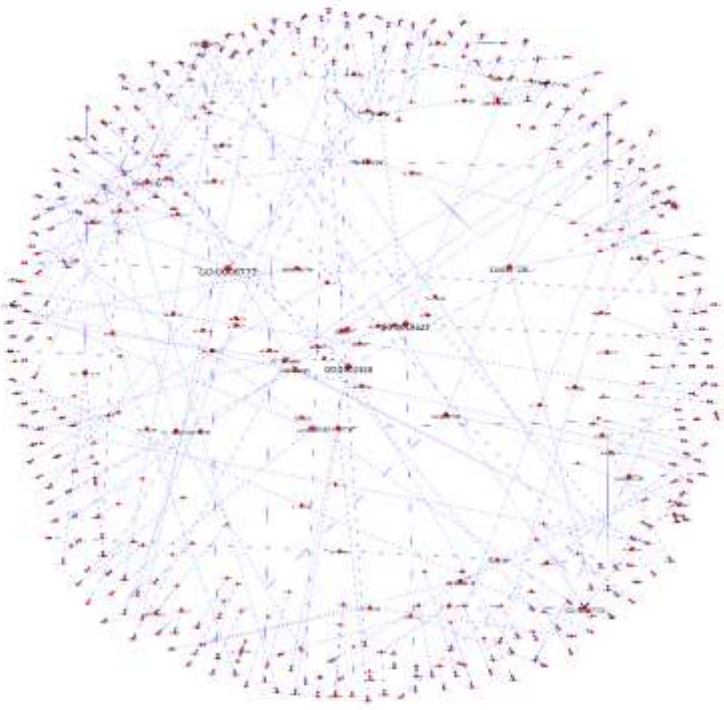

Supplementary Fig. 1 The graph of GO terms. Each GO term is represented as a node in the graph, and each edge represents a type of relationship between two GO terms.

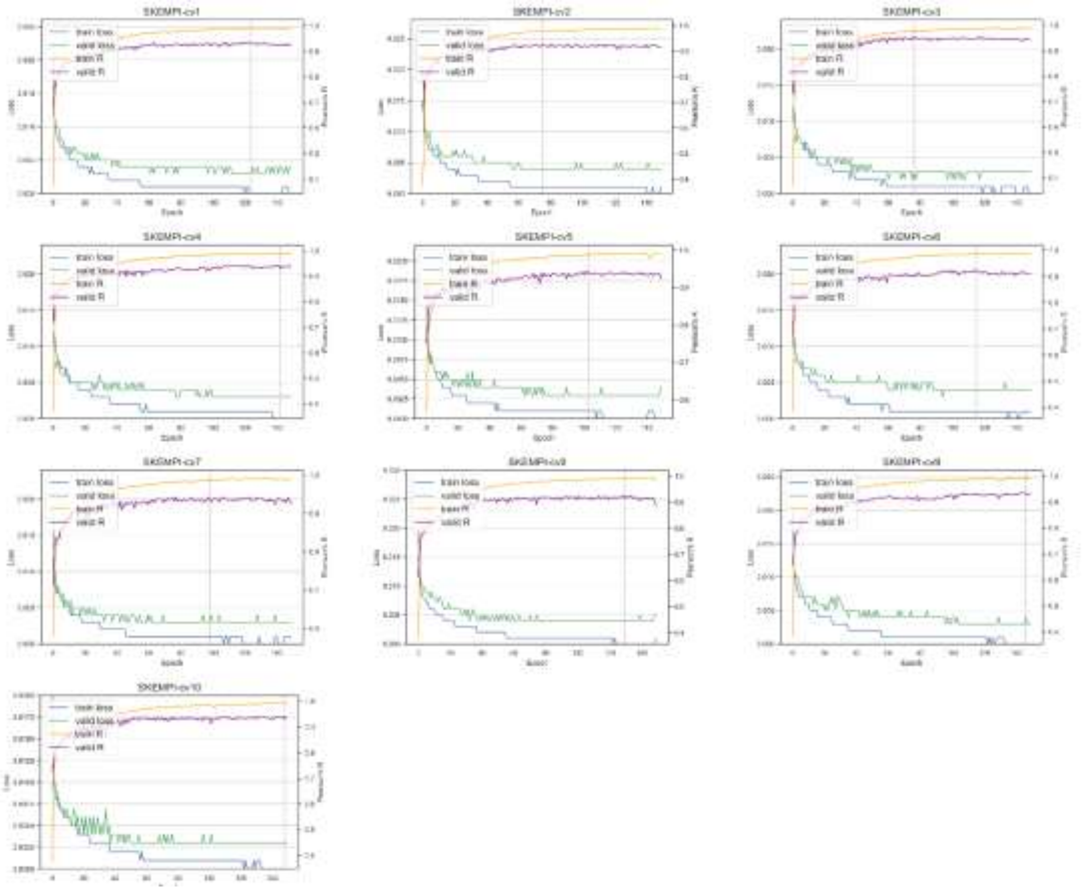

Supplementary Fig. 2 The 10-fold training processes for SKEMPI set.

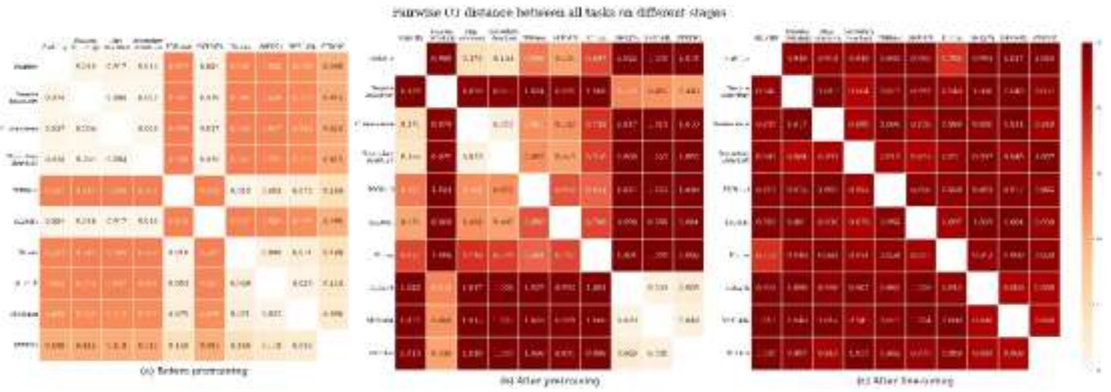

Supplementary Fig. 3 The pairwise distances of downstream tasks following pretraing-to-finetuning.

For AlphaFold2 predicted structure, it produces a per-residue confidence metric called predicted local distance difference test (pLDDT) on a scale from 0 to 100. pLDDT estimates how well the prediction would agree with an experimental structure based on the local distance difference test Cα (lDDT-Cα). A cut-off of pLDDT > 70 corresponds to a generally correct backbone prediction[1].

We have examined the accuracy of the used AlphaFold2 predicted structures. As shown in Re Fig.1a and b, 79.5% of the used predicted structures are accurate during the pretraining phase (pLDDT > 70) while 72% of predicted downstream PPI structures (SHS27k/SHS148k/STRING) are accurate (Re Fig.1c). We then investigated whether structural accuracy would impact the performance of PPI tasks. As depicted in Re Fig. 1d, the experimental group that masked low-accuracy structures achieves comparable results to the original group for both BFS and DFS splitting settings, indicating that the inclusion of low-confidence structures may not significantly affect model performance. Other modalities and the PPI graph networks may be able to correct the bias caused by low-accuracy structures.

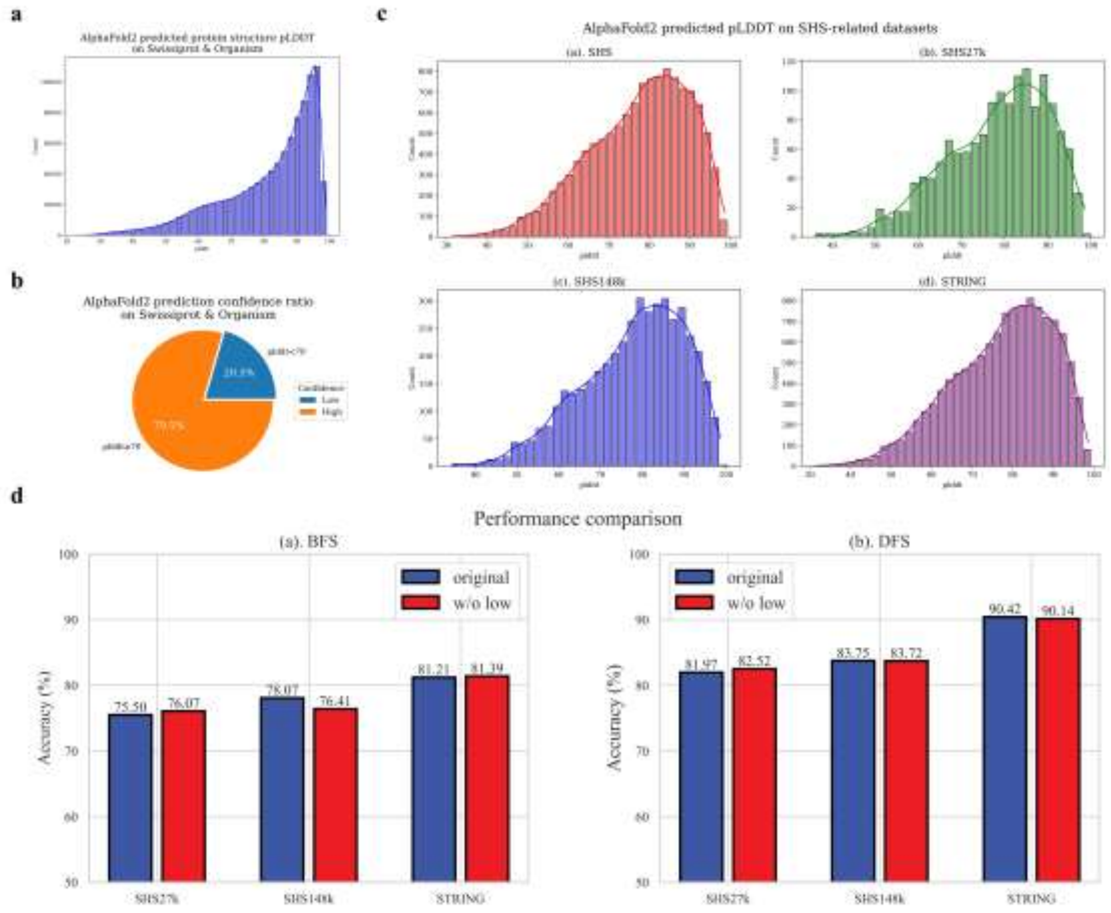

Supplementary Fig. 4 **a-c**, AlphaFold2 prediction pLDDT distribution statistical analysis. **d**, performance comparison of original group and experimental group that masks low-accuracy structure.

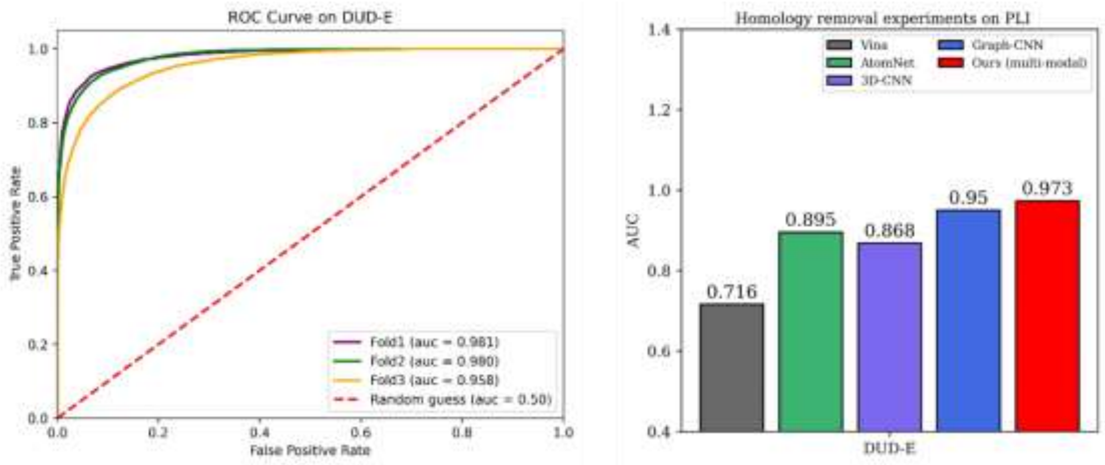

Supplementary Fig. 5 Model performance and comparison on the DUD-E, with three-fold cross-validation and random-guess ROC curves plotted in contrasting colors.

GVP module

GVP is a calculation module for multi-layer transformation of features. Given a tuple $(\boldsymbol{s}, \boldsymbol{V})$, where $\boldsymbol{s}$ is a scalar feature $\boldsymbol{s} \in \boldsymbol{R}^{s}$ and $\boldsymbol{V}$ is a geometric feature $\boldsymbol{V} \in \boldsymbol{R}^{v\times 3}$. Calculate the new feature representation$(\boldsymbol{s}', \boldsymbol{V}') \in \boldsymbol{R}^{s'} \times \boldsymbol{R}^{v'\times 3}$ following the GVP module. The detailed calculation procedure for the entire module is displayed in the table below:

Algorithm Geometric Vector Perceptrons, GVPs

Input: Scalar features: $\boldsymbol{s} \in \boldsymbol{R}^{s}$, Geometric features: $\boldsymbol{V} \in \boldsymbol{R}^{v\times 3}$

Output: Converted scalar features: $\boldsymbol{s}' \in \boldsymbol{R}^{s'}$, Converted geometric features: $\boldsymbol{V}' \in \boldsymbol{R}^{v'\times 3}$

1. $\boldsymbol{c} = \boldsymbol{max}(\boldsymbol{v}, \boldsymbol{v}')$
2. $\boldsymbol{V_c} = \boldsymbol{W_c V} \in \boldsymbol{R}^{c\times 3}$, $\boldsymbol{V_{v'}} = \boldsymbol{W_{v'} V_c} \in \boldsymbol{R}^{v'\times 3}$
3. $\boldsymbol{s_c} = ||\boldsymbol{V_c}||_2 \in \boldsymbol{R}^{c}$, $\boldsymbol{v_{v'}} = ||\boldsymbol{V_{v'}}||_2 \in \boldsymbol{R}^{v'}$
4. $\boldsymbol{s_{c+s}} = \boldsymbol{concat}(\boldsymbol{s_c}, \boldsymbol{s}) \in \boldsymbol{R}^{c+s}$
5. $\boldsymbol{s_{s'}} = \boldsymbol{W_{s'} s_{c+s}} + \boldsymbol{b} \in \boldsymbol{R}^{s'}$
6. $\boldsymbol{s}' = \boldsymbol{\sigma}(\boldsymbol{s_{s'}}) \in \boldsymbol{R}^{s'}$, $\boldsymbol{V}' = \boldsymbol{\sigma}^{+}(\boldsymbol{v_{v'}}) \odot \boldsymbol{V_{v'}} \in \boldsymbol{R}^{v'\times 3}$

Return: $(\boldsymbol{s}', \boldsymbol{V}')$

There are two primary linear transformation matrices in the calculation described above: $\boldsymbol{W_c}$ for scalar feature transformation and $\boldsymbol{W_{s'}}$ for geometric feature conversion. The subsequent two activation functions are based on L2 normal form σ and σ^{+}. In addition, before scalar feature conversion, GVP splices the feature vector $\boldsymbol{V_c}$ after geometric feature conversion is L2 regular, which can extract rotation invariance information from $\boldsymbol{V}$. GVP inserts an additional linear transformation matrix $\boldsymbol{W_{v'}}$ prior to the nonlinear transformation of geometric features, which can be separated from the extracted norm in order to control the output's dimension.

GVP-GNN is a comprehensive module comprised of GVP and GNN. The main calculation is as follow:

$$\mathrm{h}_c^{(j\to i)} = GVP\left(concat\left(\mathrm{h}_v^{(j)},\ \mathrm{h}_e^{(j\to i)}\right)\right) \quad (1)$$

$$\mathrm{h}_v^{(i)} = LayerNorm\left(\mathrm{h}_v^{(i)} + \frac{1}{k} Dropout\left(\sum_{j:e_{j\to i}\in\varepsilon} \mathrm{h}_c^{(j\to i)}\right)\right) \quad (2)$$

$$\mathrm{d}_v^{(i)} = concat(\mathrm{s}_v^{(i)}, ||\mathrm{V}_v^{(i)}||_2) \quad (3)$$

$$h'^{(i)}_v = \sigma(\boldsymbol{W}_d \mathrm{d}_v^{(i)} + \boldsymbol{b}) \quad (4)$$

where $\mathrm{h}_v^{(j)}$ and $\mathrm{h}_e^{(j\to i)}$ represents the node feature set and edge feature set of the graph, respectively. $\mathrm{h}_v^{(j)} = (\boldsymbol{s_v}, \boldsymbol{V_v}) \in \boldsymbol{R}^{m_v} \times \boldsymbol{R}^{\mu_v \times 3}$, $\mathrm{h}_e^{(j\to i)} = (\boldsymbol{s_e}, \boldsymbol{V_e}) \in \boldsymbol{R}^{m_e} \times \boldsymbol{R}^{\mu_e \times 3}$, Formula (1) outputs the intermediate feature $\mathrm{h}_c^{(j\to i)} = (\boldsymbol{s_c}, \boldsymbol{V_c}) \in \boldsymbol{R}^{m_c} \times \boldsymbol{R}^{\mu_c \times 3}$ that combines information from neighboring nodes and edges.

In formula (2), take the mean of all intermediate features, where k is the number of neighbor nodes, and then output the representation of the center node, $\mathrm{h}_v^{(i)} = (\boldsymbol{s_f}, \boldsymbol{V_f}) \in \boldsymbol{R}^{m_f} \times \boldsymbol{R}^{\mu_f \times 3}$. In Formula (3) and Formula (4), convert the feature set $(\boldsymbol{s_f}, \boldsymbol{V_f})$ into a single feature $h'^{(i)}_v \in \boldsymbol{R}^{m_\mathrm{h}}$, as the final characterization of amino acids, which is embedded in the protein structural information.

Structure and GO term mask technique

(1) For proteins lacking structure information, we assign a unified structure by setting the coordinates of all atoms to "NaN". Prior to alignment with the sequence embedding, the obtained structure features are masked following encoding by the structure encoder. We generate a mask of the same shape as the structure's features with all values set to zero, which is then used to mask the structure's features with a very small number before being passed to the softmax function. This is equivalent to generating a feature embedding with the same shape as the structure feature, but with all values set to a small constant value, such as -1×10^{-9}.

(2) For proteins lacking GO annotations, we uniformly annotate them as "No goterm," i.e., we construct a graph with a single "No goterm" node. After encoding with the GraphGO encoder and the GO encoder, we perform a mask operation on the resulting GO features before aligning and fusing them with the protein features. The alignment module is a Transformer Decoder with protein features as the source input and GO features as the target input. In the attention mechanism, the mask operation on the GO features eliminates GO information by querying the K and V of the protein features with a constant Q.

By utilizing the above processing and operations for structure and GO annotations, our model can avoid the need for structure and GO annotations in the embedding generation, allowing it to perform tasks, such as protein property prediction, that require only sequence inputs.

Ablation study on homologous proteins

In order to make fair comparisons with other landmark methods, the train/valid/test sets of all protein property datasets were obtained from their original sources (TAPE[2]: github.com/songlab-cal/tape, ProteinBert[3]: github.com/nadavbra/protein_bert). The samples within the train, valid, and test sets were identical with other methods

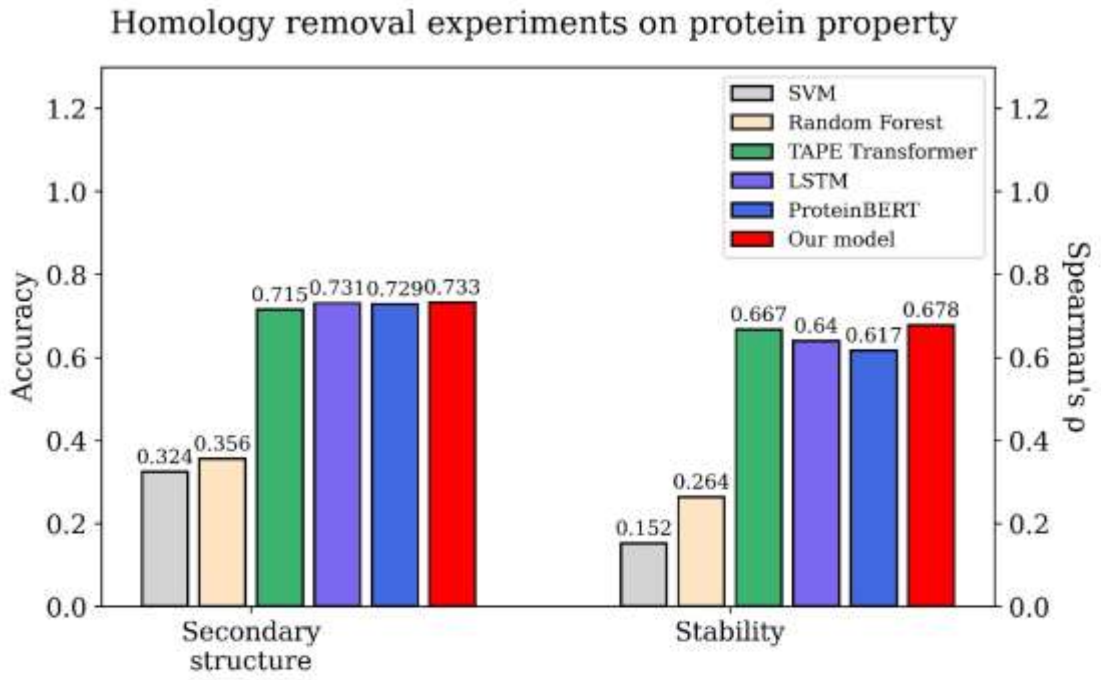

Supplementary Fig. 6 Protein sequence homology ablation experiments (stability and secondary structure).

Supplementary Table 1. Protein sequence homology ablation experiments (PPI-BFS)

**Method**	**SHS27k (accuracy)**	**SHS148k (accuracy)**	**STRING (accuracy)**
MASSA	71.83	57.74	64.43
PIPR	37.53	47.83	41.55
GNN-PPI	60.21	54.42	61.60
ProBERT	69.67	52.33	62.22
OntoProtein	67.37	54.82	63.65

Statistical significance between MASSA and ProteinBERT

We have examined the statistical significance between MASSA and ProteinBERT with regard to the four protein property benchmarks. Specifically, for each protein property task, we randomly sampled the test results of MASSA and ProteinBERT 10 times, selecting approximately 20% of the samples each time, and then computed the corresponding evaluation indicators. MASSA, proteinBERT, and the evaluation index of each sample were used as observation variables and observation values, respectively. The Independent Sample t-test was employed to determine whether the difference between the two was statistically significant. As shown in Re Fig. 6, the statistic p value is less than 0.05 for both stability and fluorescence tasks, indicating that the difference between MASSA and ProteinBERT is statistically significant. In contrast, the statistical p value is greater than 0.05 for the secondary structure and remote homology tasks, indicating that the difference between MASSA and PorteinBERT is not statistically significant.

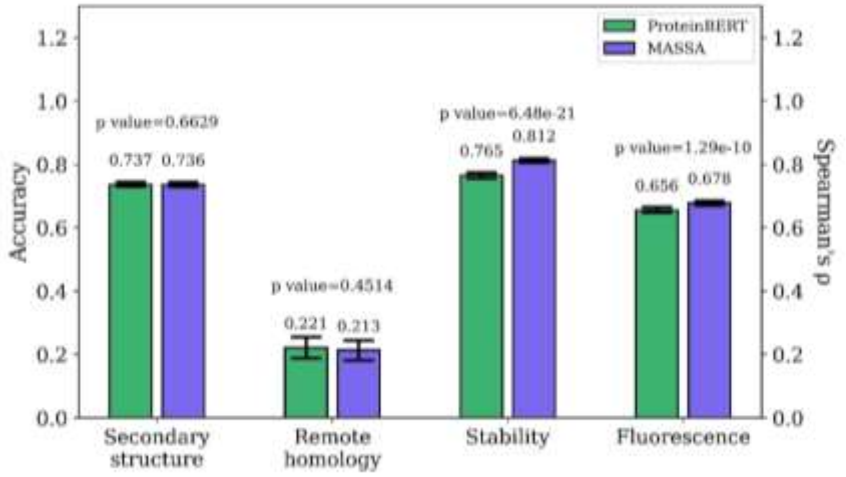

Supplementary Fig. 7 **Test for statistical significance (MASSA and ProteinBERT)**

Pretraining details

A server with four NVIDIA GeForce RTX 3090 GPUs was used to pretrain the MASSA for 150 epochs over the course of 32 days. On the same server, the fine-tuning operations took one to three days, depending on the downstream task. It was done using the following hyperparameters: RAdam[4] optimizer with $\beta_1 = 0.9, \beta_2 = 0.999, \epsilon = 10^{-8}$ and $learning\_rate = 10^{-4}, weight\_decay = 10^{-4}$. In addition, we employed the Lookahead[5] optimization approach with k=5,=0.5.

The comparison of transferability metrics

A significant advantage of optimal-transport(OT) over other approaches is that it permits efficient and accurate comparison of distributions with little or no intersection, which is a common scenario in bioinformatics involving heterogeneous tasks.

In accordance with your suggestion, we compare OT's trade-off between time cost and performance to four approaches, including Euclidean distance, cosine distance, H-score[6], and LogMe[7]. As depicted in Re Fig. 8, the computation time of all approaches increases quadratically with the size of the set, whereas OT achieves significant performance improvements at less than double the cost in time. Specifically, the matrix comparison-based methods, Euclidean distance and cosine distance, require approximately half the time of OT, but their performance is significantly diminished. H-score and LogMe, which were proposed to deal with inter-task transferability in the CV field, fail in the complex scenario of heterogeneous tasks in bioinformatics. OT strikes a better balance between time cost and performance, making it the superior option.

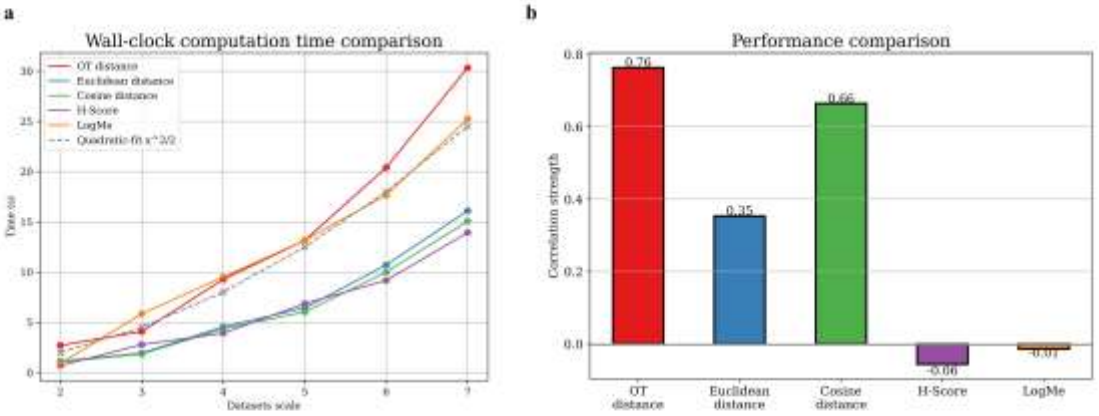

Supplementary Fig. 8 The comparison of transferability metrics.

Extended Data Figures

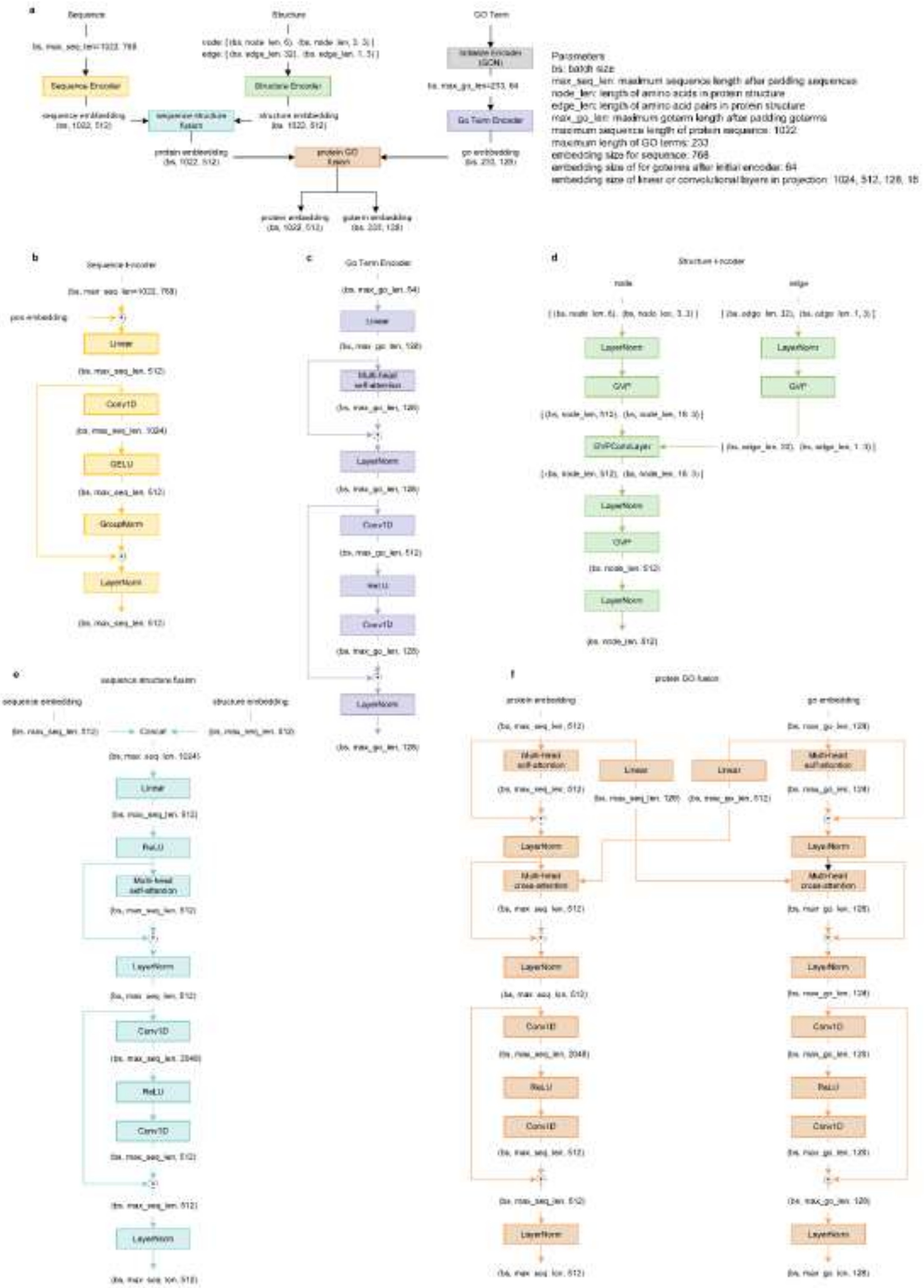

Extended Data Fig. 1 | Submodules of the proposed MASSA model. a, General overview of the multimodal fusion process. **b,** Sequence encoder for extracting sequence features. **c,** GO term encoder for extracting GO features. **d,** Structure encoder for extracting structure features. **e,** The

fusion of sequence and structure features. **f,** The fusion of protein (sequence–structure) and GO features.

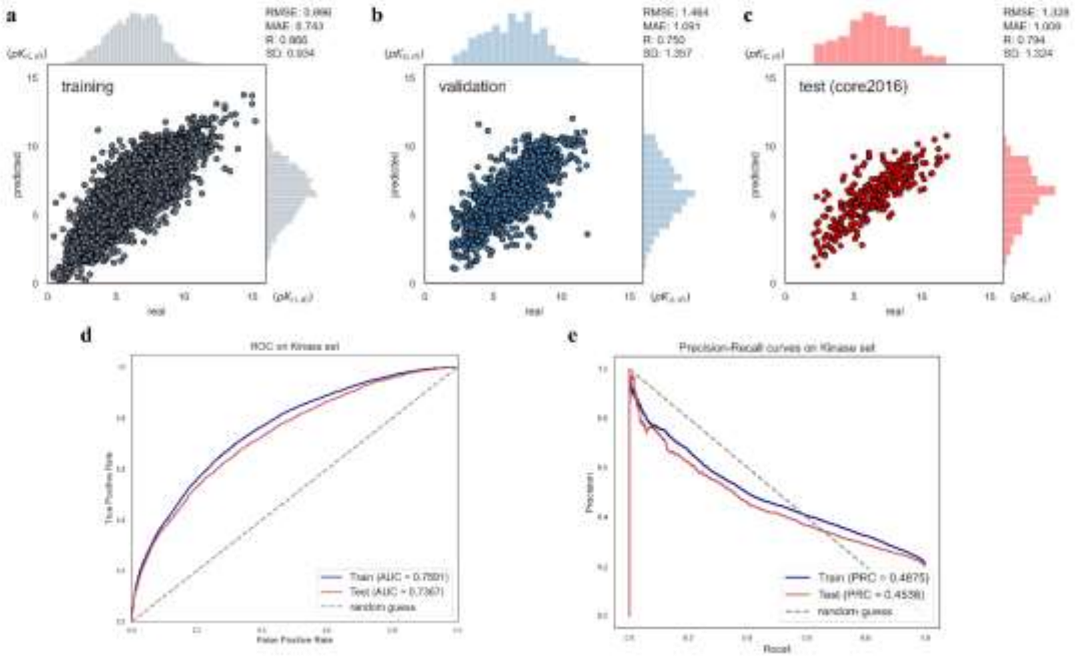

Extended Data Fig. 2 | Model performance on PLI benchmarks. a-c, Training, validation and test (core2016) performance on PDBbind v.2016. **d,** ROC curves of the training and test sets on the Kinase set. **e,** Precision–recall curves of the training and test sets on the Kinase set.

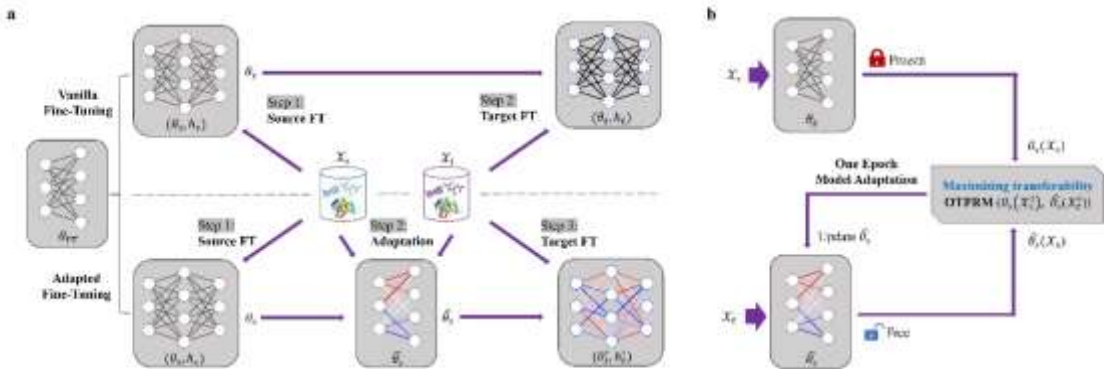

Extended Data Fig. 3 | OTFRM-guided fine-tuning strategy. a, Procedures of Vanilla-FT and Adapted-FT. **b,** Detailed steps of model adaptation based on the OTFRM.

Reference

[1] J. Jumper, R. Evans, A. Pritzel, T. Green, M. Figurnov, O. Ronneberger, K. Tunyasuvunakool, R. Bates, A. Žídek, A. Potapenko, A. Bridgland, C. Meyer, S. A. A. Kohl, A. J. Ballard, A. Cowie, B. Romera-Paredes, S. Nikolov, R. Jain, J. Adler, T. Back, S. Petersen, D. Reiman, E. Clancy, M. Zielinski, M. Steinegger, M. Pacholska, T. Berghammer, S. Bodenstein, D. Silver, O. Vinyals, A. W. Senior, K. Kavukcuoglu, P. Kohli, D. Hassabis, *Nature* **2021**, *596*, 583.

[2] R. Rao, N. Bhattacharya, N. Thomas, Y. Duan, X. Chen, J. Canny, P. Abbeel, Y. S. Song, *Proc. Adv. Neural Inf. Process. Syst.* **2019**, 9689.

[3] N. Brandes, D. Ofer, Y. Peleg, N. Rappoport, M. Linial, *Bioinformatics* **2022**, *38*, 2102.

[4] L. Liu, H. Jiang, P. He, W. Chen, X. Liu, J. Gao, J. Han, in *Proc. Eighth Int. Conf. Learn. Represent. (ICLR 2020)*, **2020**, pp. 1–13.

[5] M. R. Zhang, J. Lucas, G. Hinton, J. Ba, *arxiv* **2019**, 1.

[6] Y. Bao, Y. Li, S.-L. Huang, L. Zhang, L. Zheng, A. Zamir, L. Guibas, in *2019 IEEE Int. Conf. Image Process.*, IEEE, **2019**, pp. 2309–2313.

[7] K. You, Y. Liu, J. Wang, M. Long, *ICML* **2021**.
